# Klotho Regulated by Estrogen Plays a Key Role in Sex Differences in Stress Resilience in Rats

**DOI:** 10.3390/ijms24021206

**Published:** 2023-01-07

**Authors:** Zhinei Tan, Yongxia Li, Yinzheng Guan, Javed Iqbal, Chenyue Wang, Riqiang Yan, Xin-Ming Ma

**Affiliations:** 1 College of Life Sciences, Shaanxi Normal University, Xi’an 710119, China; 2 Department of Neuroscience, University of Connecticut Health, Farmington, CT 06030, USA

**Keywords:** hippocampus, stress, CUMS, learning and memory, anhedonia, anxiety, estradiol, stress susceptibility, ovariectomy

## Abstract

Klotho (KL) is a glycosyl hydrolase and aging-suppressor gene. Stress is a risk factor for depression and anxiety, which are highly comorbid with each other. The aim of this study is to determine whether KL is regulated by estrogen and plays an important role in sex differences in stress resilience. Our results showed that KL is regulated by estrogen in rat hippocampal neurons in vivo and in vitro and is essential for the estrogen-mediated increase in the number of presynaptic vesicular glutamate transporter 1 (Vglut1)-positive clusters on the dendrites of hippocampal neurons. The role of KL in sex differences in stress response was examined in rats using 3-week chronic unpredictable mild stress (CUMS). CUMS produced a deficit in spatial learning and memory, anhedonic-like behaviors, and anxiety-like behaviors in male but not female rats, which was accompanied by a reduction in KL protein levels in the hippocampus of male but not female rats. This demonstrated the resilience of female rats to CUMS. Interestingly, the knockdown of KL protein levels in the rat hippocampus of both sexes caused a decrease in stress resilience in both sexes, especially in female rats. These results suggest that the regulation of KL by estrogen plays an important role in estrogen-mediated synapse formation and that KL plays a critical role in the sex differences in cognitive deficit, anhedonic-like behaviors, and anxiety-like behaviors induced by chronic stress in rats, highlighting an important role of KL in sex differences in stress resilience.

## 1. Introduction

The hippocampus is a primary target of estrogens and plays a key role in learning and memory, and stress responses [1]. 17β-Estradiol (E2), a major form of estrogen, plays an important role in spine formation, synaptic plasticity, and learning and memory [1,2,3]. However, the underlying mechanism is largely unknown. Our RNA-seq study showed that Klotho (KL) mRNA levels in the hippocampus are positively correlated with the levels of circulating E2 during the estrous cycle and that the OVX-mediated decrease in hippocampal KL mRNA levels is reversed by E2 replacement [4]. These results spurred an interest in further studying the regulation of KL by estrogen and the function of KL protein in the rat hippocampus. 

KL is expressed in the hippocampus [5] and exists in three different forms in humans and mice: a full-length, transmembrane protein (130 kDa); a shed form (cleavage form);and a secreted protein (70 kDa) [6,7,8,9]. Since the secreted KL is not expressed in rats [10,11], the KL we investigated in the rat hippocampus is the full-length form of KL. KL deficiency shortens the lifespan, while KL overexpression slows the aging process and extends the lifespan [8,12]. KL is localized in both pre- and post-synaptic compartments of CA1 pyramidal neurons and regulates synaptic plasticity [5]. KL-mutant mice show a decrease in both the synapse number and the level of synaptophysin in the hippocampus [13] and cognitive impairment [14]. KL-overexpressing mice show enhanced cognitive function [15]. In vitro studies show the neuroprotective properties of KL on hippocampal neurons [16]. These studies exemplify the role of KL in cognition and neuroprotection in mice. However, the functions of KL in the rat brain are not well understood.

Clinical evidence shows that low levels of circulating KL are related to depression [17,18,19]. Chronic psychological stress causes a decrease in serum KL in humans, and KL levels are positively correlated with the severity of depression symptoms [17]. Patients with major depression have decreased KL in the cerebrospinal fluid [18]. These studies suggest an important role of KL in depression. Preclinical studies show that chronic unpredictable stress results in a decrease in KL mRNA levels in the rat choroid plexus [20], and KL in the mouse nucleus accumbens regulates behavioral responses to social defeat stress [21]. These studies suggest an important role of KL in stress response. Stress is a risk factor for depression and anxiety [22], in which there is a sex difference [23]. Depression and anxiety are often present as comorbid disorders [24]. Chronic unpredictable mild stress (CUMS), an established animal model of depression, induces anhedonic-like and anxiety-like behaviors [25]. There are sex differences in stress resilience in rodents in which females generally show resilience to the detrimental effects of chronic stress on dendritic spines, synaptic plasticity, and cognition when compared with males [26,27,28,29]. However, little is known about the underlying mechanisms. These studies motivated us to determine the role of KL in sex differences in stress resilience. Furthering the knowledge of stress resilience should result in a finer understanding of the mechanisms underlying stress-related disorders. Our hypothesis is that KL protein levels in the rat hippocampus are regulated by estrogen, which contributes to the mechanisms underlying estrogen-mediated synapse formation and that KL plays a key role in CUMS-induced sex differences in cognitive deficit, anhedonic-like behaviors, and anxiety-like behaviors in rats. Cognitive deficit and anhedonia, which are the core future of depression as well as anxiety, are aligned with important Research Domain Criteria (RDoC) [30]. Our aim was to test our hypothesis, and the results from this study will likely enhance our understanding of estrogen-mediated synapse formation and sex differences in stress resilience.

## 2. Results

### 2.1. E2 Regulated the KL Protein Levels in the Hippocampal Neurons

**Figure 1:**
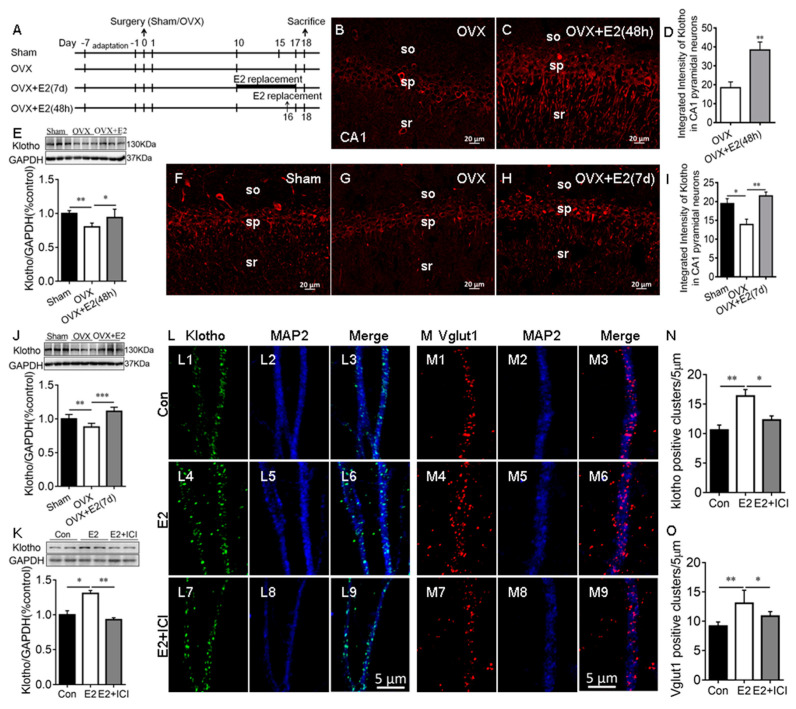
E2 increased Klotho expression in the hippocampus of ovariectomized (OVX) rats and cultured hippocampal neurons. Experimental design (**A**). Representative images of KL immunostaining (red) in the hippocampal CA1 area of sham, OVX, and E2-treated OVX (OVX + E2) rats (48 h after vehicle or E2 injection (**B**,**C**); daily vehicle or E2 injection for 7 days (**F**–**H**)). Quantification of fluorescence intensity of KL staining in the hippocampal CA1 area (48 h (**D**); 7 d (**I**)). Western blot showing the relative KL protein levels in the hippocampus of sham, OVX, and OVX + E2 rats (48 h (**E**); 7 d (**J**); n = 6). The cultures were treated with the vehicle (Con), 10 nM E2, and E2 + 1 μM ICI (ICI182,780, estrogen receptor inhibitor) at Div13 for 48 h. Western blot result of hippocampal lysate prepared from primary hippocampal culture (**K**). Immunostaining of cultured hippocampal neurons with antibodies specific to KL and MAP2 in Con, E2, and E2 + ICI neurons (**L**). KL staining (green, (**L1**,**L4**,**L7**)), MAP2 staining (blue, (**L2**,**L5**,**L8**)), and merge of KL and MAP2 (**L3**,**L6**,**L9**). Quantification of KL-positive clusters in cultured hippocampal neurons (**N**). Immunostaining of cultured hippocampal neurons with antibodies specific to Vglut1 and MAP2 in Con, E2, and E2 + ICI neurons (**M**). Vglut1 staining (red, (**M1**,**M4**,**M7**)), MAP2 staining (blue, (**M2**,**M5**,**M8**)), and merge of Vglut1 and MAP2 (**M3**,**M6**,**M9**). The number of Vglut1-positive clusters in cultured hippocampal neurons (**O**). SO, stratum oriens of CA1 area; SP, stratum pyramidale of CA1 area; SR, stratum radiatum of CA1 area; Con, control. Data are shown as the mean ± SEM. D with the *t*-test, and others with one-way ANOVA followed by Tukey’s test. * *p* < 0.05, ** *p* < 0.01, *** *p* < 0.001.

Our RNA-seq study showed that E2 upregulates KL mRNA levels in the rat hippocampus [4]. To confirm this result, OVX rats received vehicle or E2 treatments for 48 h and 7 days (Figure 1A). Immunostaining results showed that KL is localized in the soma and dendrites of the hippocampal CA1 pyramidal neurons of rats, as expected (Figure S1) [5,31] and the antibody specificity is validated in KL-knockout mice (Figure S1). E2 treatment for 48 h significantly increased the levels of KL staining intensity in the hippocampal CA1 pyramidal neurons of the OVX + E2 group compared to the vehicle-treated OVX group (*t*_12_ = 3.78, *p* = 0.003; Figure 1B–D). Western blot analysis showed that 48 h E2 treatment has a significant effect on KL protein levels in the hippocampus (*F*_2, 15_ = 9.18, *p* = 0.003; Figure 1E). Similarly, 7-day E2 treatment caused a significant increase in the levels of KL staining intensity in the hippocampal CA1 pyramidal neurons (*F*_2, 18_ = 9.5, *p* = 0.002; Figure 1F–I) and KL protein levels in the hippocampus (*F*_2, 15_ = 22.21, *p* < 0.001; Figure 1J). These results confirmed that the OVX-induced decrease in KL expression is reversed by E2 treatment. To determine the effects of E2 on KL and Vglut1 expression in cultured hippocampal neurons, primary cultures were treated with the vehicle (Figure 1(L1–L3,M1–M3)), E2 (Figure 1(L4–L6,M4–M6)), or E2 + ICI182,780 (Figure 1(L7–L9,M7–M9)) at Div13 for 48 h, as described in our previous study [32]. Vglut1, a marker for excitatory presynaptic terminals, was upregulated by E2 treatment, and Vglut1-positive cluster numbers were used to evaluate the number of excitatory presynaptic terminals along MAP2-positive dendrites (a positive control for KL) [32,33]. E2 treatment resulted in a significant increase in KL protein levels (*F*_2, 6_ = 38.86, *p* = 0.007; Figure 1K), which was determined by Western blot and the number of KL positive clusters along MAP2-positive dendrites (*F*_2, 21_ = 10.65, *p* = 0.004; Figure 1N). The E2-induced increase in both KL protein levels and the number of KL-positive clusters was reversed by ICI182,780 treatments (Figure 1K–N).

As expected, E2 had a significant effect on the number of Vglut1-positive clusters along MAP2-positive dendrites (*F*_2, 15_ = 11.15, *p* = 0.001; Figure 1O). The E2-induced increase in the number of Vglut1-positive clusters (*p* < 0.01; Figure 1(M4–M6,O)) was reversed by ICI182,780 (*p* < 0.05; Figure 1(M7–M9,O)). In addition, the inhibition of endogenous E2 synthesis with letrozole caused a decrease in KL protein levels, KL-positive clusters, and Vglut1-positive clusters along MAP2-positive dendrites in the hippocampal neurons (Figure S2) [34]. Overall, these results showed that E2 regulates the levels of KL protein in rat hippocampal neurons. To determine whether E2 regulates KL expression in the hippocampus of male rats, male rats received vehicle or E2 treatment for 7 days, and Western blot analysis showed that E2 treatment increases KL protein levels in the male hippocampus (Figure S3).

### 2.2. E2 Did Not Affect the Number of Vglut1-Positive Excitatory Presynaptic Terminal When the Endogenous KL Level Was Reduced in Hippocampal Neurons

**Figure 2:**
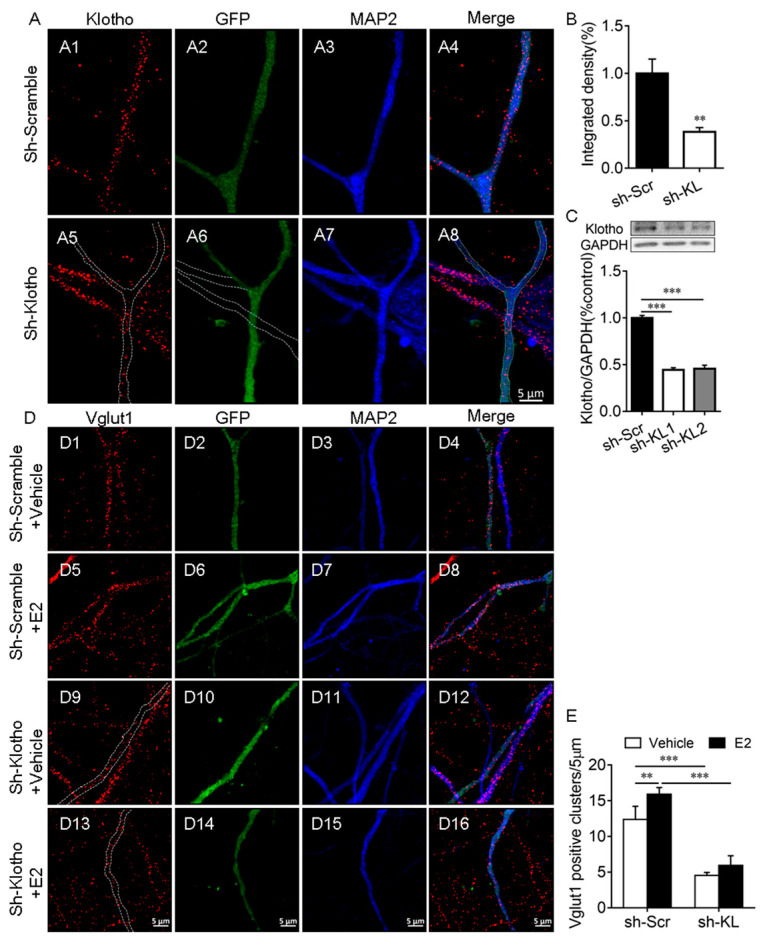
Klotho played an essential role in the E2-mediated increase in the number of Vglut1-positive clusters in hippocampal neurons. Primary cultured hippocampal neurons were transfected with a vector encoding scrambled control shRNA (sh-Scr, (**A1**–**A4**)) or Klotho shRNA (sh-KL, (**A5**–**A8**)) at Div10, and the neurons were fixed for double-immunostaining with anti-KL (red) and anti-MAP2 (blue) antibodies at Div14. The dashed lines in (**A6**) show the dendrites of the non-transfected neuron, which did not express Klotho shRNA-GFP (**A**). Quantification of KL expression in cultured hippocampal neurons (**B**). Two different Klotho shRNAs, sh-KL #1 and sh-KL# 2, were designed. Vectors encoding sh-Scr and sh-KL were introduced into the hippocampal neurons by electroporation at the time of plating, and the Western blot result (**C**) showed that the expression of shRNA #1 and #2 reduces klotho (130 kDa) expression effectively. We used sh-KL #1 in subsequent experiments. Cultured hippocampal neurons were transfected with a vector encoding sh-Scr (**D1**–**D8**) and sh-KL (**D9**–**D16**) at Div10, and the cultures at Div13 were treated with the vehicle (**D1**–**D4**, **D9**–**D12**) or 10 nM E2 (**D5**–**D8**, **D13**–**D16**) for 48 h before fixing for double-immunostaining with antibodies specific to Vglut1 (red) and MAP2 (blue; **D**). Quantification of Vglut1-positive clusters (**E**). One-way and two-way ANOVA followed by Tukey’s test. Data are shown as the mean ± SEM. ** *p* < 0.01, *** *p* < 0.001.

This experiment was to determine whether E2 affects the number of Vglut1-positive clusters along dendrites when endogenous KL protein levels reduced. First, we demonstrated that the expression of KL-shRNA1 causes a significant decrease in both the intensity of KL staining (*t*_12_ = 3.91, *p* = 0.002; Figure 2A,B) and the levels of KL protein in cultured hippocampal neurons (*F*_2, 9_ = 275.8, *p* < 0.001; Figure 2C).

To determine whether the E2-mediated upregulation of KL expression is required for the E2-induced increase in the number of Vglut1-positive clusters, cultured hippocampal neurons were transfected with a vector encoding KL-shRNA-GFP or scrambled shRNA-GFP at Div10. At Div13, the cultures were treated with the vehicle or E2 for 48 h (Figure 2D), as described previously [32]. Two-way ANOVA showed that KL-shRNA and E2 treatment have a significant effect on the number of Vglut1-positive clusters (interaction: *F*_1, 21_ = 4.71, *p* = 0.04; shRNA: *F*_1, 21_ = 324.8, *p* < 0.001; E2: *F*_1, 21_ = 24.76, *p* < 0.001; Figure 2D,E). Post hoc Tukey’s test showed that in KL-shRNA-expressing neurons, Vglut1-positive clusters decrease in comparison to scrambled-shRNA-expressing2 neurons (*p* < 0.001; Figure 2(D9–D12) vs. (D1–D4), Figure 2E). In scrambled-shRNA-expressing neurons, E2 treatment caused a significant increase in the number of Vglut1-positive clusters versus vehicle-treated scrambled-shRNA-expressing neurons (*p* < 0.01; Figure 2(D5–D8) vs. (D1–D4), Figure 2E). However, this effect of E2 was absent in KL-shRNA-expressing neurons (*p* > 0.05; Figure 2(D13–D16) vs. (D9–D12), Figure 2E). E2 no longer increased the number of Vglut1-positive clusters in hippocampal neurons after KL expression decreased due to KL-shRNA.

### 2.3. CUMS-Induced Deficit in Spatial Learning and Memory, Anhedonic-like Behaviors, and Anxiety-like Behaviors Were Accompanied by a Decrease in KL Protein Levels in Male Rats Only

**Figure 3:**
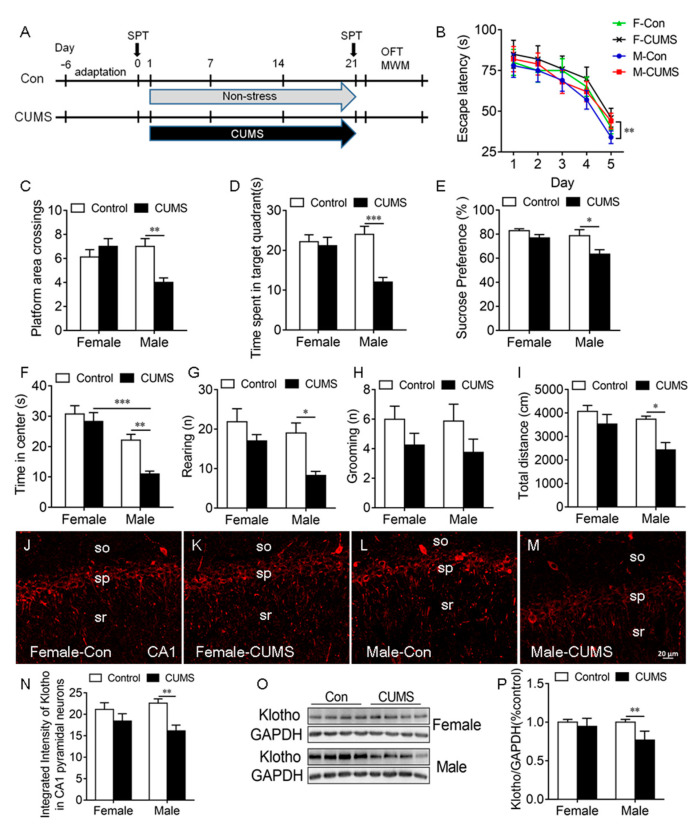
CUMS-induced spatial learning and memory impairment, anhedonic-like behaviors, and anxiety-like behaviors in male rats were accompanied by a decrease in KL protein levels in the hippocampus of male but not female rats. Experimental design. Sucrose preference test (SPT), open-field test (OFT), and Morris water maze (MWM) test (**A**). Latency to the platform in rats of both sexes during 5-day training in the MWM test. CUMS decreased the latency on day 5 during training in male but not female rats (**B**). CUMS decreased the number of platform crossings (**C**) and the time spent in the target quadrant (**D**) in males only on day 6 during the probe trial. Only male rats showed a decrease in sucrose consumption in the SPT after CUMS (**E**). Time spent in the center of the open field (**F**), the number of rearing times (**G**), the number of grooming times (**H**), and the total distance traveled (**I**) in the OFT. Representative confocal images of KL staining (red) in the hippocampal CA1 area in female control (**J**), female CUMS (**K**), male control (**L**), and male CUMS (**M**) rats. Quantification of fluorescence intensity of KL staining in the hippocampal CA1 area in male and female rats (**N**). Western blot analysis of KL protein (130 kDa) in the hippocampus of male and female rats (**O**,**P**). Data are shown as the mean ± SEM. Two-way ANOVA followed by Tukey’s test. * *p* < 0.05, ** *p* < 0.01, *** *p* <0.001, n = 8–10.

To determine the effects of CUMS on KL protein expression in the rat hippocampus, rats of both sexes received CUMS for 3 weeks (Figure 3A).

Spatial learning and memory. The Morris water maze (MWM) test was performed to evaluate spatial learning and memory. On day 5 during the 5-day learning phase, we detected a significant main effect of stress (*F*_1, 28_ = 23.51, *p* < 0.0001) and sex (*F*_1, 28_ = 5.88, *p* = 0.022) on the escape latency to the platform, where only male stressed rats took a significantly longer time to find the platform compared to unstressed males (*p* < 0.01; Figure 3B). The stress × sex interaction did not have a significant effect (*F*_1,28_ = 1.47, *p* = 0.24).

During the probe trial on day 6, stress and sex had a significant effect on platform area crossings (interaction: *F*_1, 28_ = 10.94, *p* = 0.003; Figure 3C, Table S1) and the time spent in the target quadrant (interaction: *F*_1, 28_ = 9.29, *p* = 0.005; stress: *F*_1, 28_ = 12.97, *p* = 0.001; sex: *F*_1, 28_ = 4.03, *p* = 0.05; Figure 3D). Tukey’s test showed that stressed male but not stressed female rats exhibited a decrease in platform area crossings (*p* < 0.01; Figure 3C) and the time spent in the target quadrant (*p* < 0.001; Figure 3D) compared to unstressed controls (Figure 3B–D). 

Although the MWM test has been widely used to evaluate CUMS-induced deficits in spatial learning and memory [35,36,37,38], swimming in the MWM may be a stressor to animals and affect the CUMS-mediated results. The Barnes maze test (BMT) is generally thought to be less stressful than the Morris water maze test according to plasma corticosterone levels and more sensitive to early cognitive deficits [39,40]. To determine whether the swimming in the MWM affects the results of the MWM test, another cohort of rats of both sexes received the same CUMS exposure and the BMT was performed to verify the results of the MWM test. Our results showed that swimming in the MWM does not affect the results of the MWM test, since the results of the MWM test and the BMT were identical (Figure S4), which may result from the fact that the swimming included in our CUMS protocol was a novel stress to rats in the MWM test.

In the Barnes maze test, stress and sex displayed a significant effect on the escape latency on day 4 during the 4-day training (interaction: *F*_1, 24_ = 9.67, *p* = 0.005; sex: *F*_1, 24_ = 15.99, *p* = 0.0005; stress: *F*_1, 24_ = 33.37, *p* < 0.0001; Figure S4A). Tukey’s test showed that only stressed males exhibited an increase in the escape latency compared to unstressed males (*p* < 0.0001). On day 7 during the probe trial, there was a significant effect of sex and/or stress on the escape latency (interaction: *F*_1, 24_ = 8.71, *p* = 0.007; stress: *F*_1, 24_ = 22.99, *p* < 0.0001; Figure S4B, Table S1). Only stress had a significant effect on the number of errors to find the target hole (*F*_1, 24_ = 20.59, *p* = 0.0001) and the time spent in the target quadrant (*F*_1, 24_ = 8.32, *p* = 0.008). Tukey’s test showed that on day 7 during the probe trail, stressed males but not stressed females showed an increase in the escape latency (*p* < 0.001; Figure S4B), a decrease in time spent in the target quadrant (*p* < 0.05; Figure S4C), and an increase in number of errors to locate the target hole (*p* < 0.001; Figure S4D) compared to unstressed controls. These results showed that CUMS causes a deficit in spatial learning and memory in males only and that females are resilient to CUMS.

Anhedonic-like behavior. Sucrose consumption in the SPT was used to evaluate anhedonic-like behavior. Stressed rats that did not like sweetness drank less sucrose solution and showed anhendoic-like behavior. Two-way ANOVA revealed a significant main effect of sex (*F*_1, 36_ = 6.14, *p* = 0.02) and stress (*F*_1, 36_ = 9.15, *p* = 0.005) but not of the sex × stress interaction (*F*_1, 36_ = 1.76, *p* = 0.19) on sucrose consumption ((Table S1). CUMS caused a significant decrease in sucrose consumption in males compared to male controls (*p* < 0.05), but CUMS did not alter sucrose consumption in females (Figure 3E). These findings showed that CUMS-induced anhedonic-like behavior is sex dependent.

Anxiety-like behavior: Stress had a significant effect on the time spent in the center (*F*_1, 36_ = 8.89, *p* = 0.006), rearing (*F*_1, 28_ = 11.39, *p* = 0.002), and the distance traveled (*F*_1, 28_ = 9.98, *p* = 0.004) in the OFT. Sex also had a significant effect on the time spent in the center (*F*_1, 28_ = 32.05, *p* < 0.0001), rearing (*F*_1, 28_ = 6.31, *p* = 0.002), and the distance traveled (*F*_1, 28_ = 5.96 *p* = 0.02) in the OFT (Table S1). Tukey’s test showed that CUMS caused a significant decrease in the time spent in the center (*p* < 0.01; Figure 3F), the number of rearing times (*p* < 0.05; Figure 3G), and the distance traveled (*p* < 0.05; Figure 3I) in the OFT in males compared to unstressed male controls. CUMS did not alter grooming in rats of both sexes (Figure 3H). These results showed that CUMS induces anxiety-like behaviors in male but not female rats.

Since KL is associated with cognition and stress response, the next experiment was to determine whether the CUMS-induced deficit in spatial learning and memory, anhedonic-like behavior, and anxiety-like behavior in male rats is accompanied by an alteration in KL expression in the hippocampus. The results showed that there is a significant main effect of stress on the integrated intensity of KL staining in CA1 pyramidal neurons (*F*_1, 16_ = 17.83, *p* = 0.006; Table S1). CUMS induced a significant decrease in the integrated intensity of KL staining in CA1 pyramidal neurons in male but not female rats compared to unstressed controls (*p* < 0.01; Figure 3J–N). Western blot results showed that stress and sex have a significant effect on the levels of KL protein in the hippocampus (interaction: *F*_1, 24_ = 7.13, *p* = 0.01; sex: *F*_1, 24_ = 7.13, *p* = 0.01; stress: *F*_1, 24_ = 18.16, *p* = 0.0003; Figure 3O,P). Tukey’s test showed that stressed male but not female rats displayed a significant decrease in the levels of KL protein in the hippocampus when compared with unstressed controls (*p* < 0.01). Females showed a significantly higher degree of resilience to stress than males. These results showed that sex differences in the CUMS-mediated decrease in KL expression may contribute to the sex differences observed in stress resilience.

### 2.4. Reduction in Endogenous KL in the Hippocampus Did Not Alter Spatial Learning and Memory, Anhedonic-like Behavior, and Anxiety-like Behavior in Rats of Both Sexes

**Figure 4:**
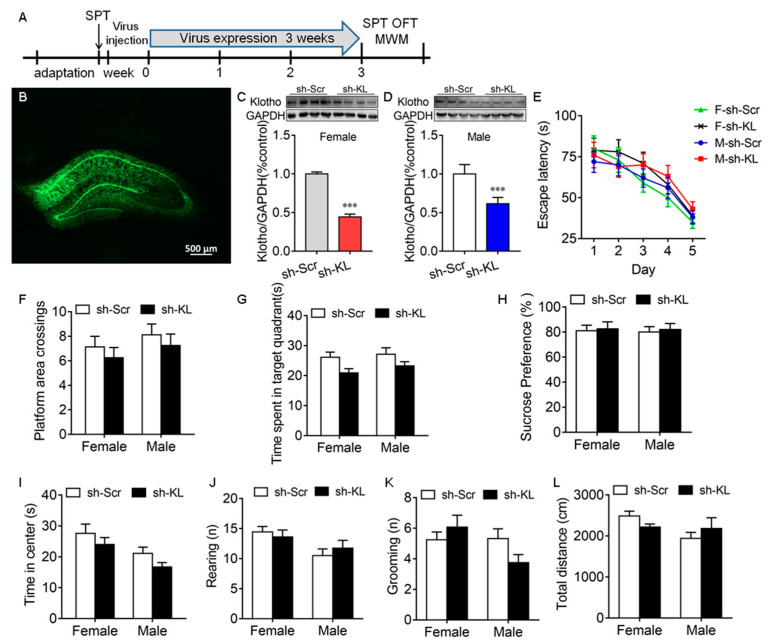
AAV-mediated decrease in the expression of hippocampal klotho (KL) protein did not impair spatial learning and memory in the Morris water maze test and induce anhedonic-like behaviors. Experimental design. Sucrose preference test (SPT), open-field test (OFT), and Morris water maze (MWM) test (**A**). Representative image of AAV-KLshRNA-GFP expression (**B**). Western blot analysis showed a decrease in the levels of hippocampal KL protein (130 kDa) due to Klotho shRNA (sh-KL) compared to scrambled shRNA (sh-Scr) in female (**C**) and male (**D**) rats. In the MWM test, the decreased expression of KL did not alter the latency to the platform on days 1–5 during training in the MWM (**E**), the number of platform area crossings (**F**), and the time spent in the target quadrant on day 6 (**G**) during the probe trial in male and female rats. No significant difference between the sh-KL and sh-Scr groups in sucrose consumption was found in the SPT (**H**). Time spent in the center of the open field (**I**), the number of rearing times (**J**), the number of grooming times (**K**), and the total distance traveled (**L**) in the OFT. (**C**,**D**) *t*-test; (**E**) two-way repeated-measures ANOVA; (**F**–**L**) two-way ANOVA followed by Tukey’s test. Data are shown as the mean ± SEM. *** *p* < 0.001, n = 8–13.

The CUMS-induced decrease in KL expression in the hippocampus was accompanied by a deficit in spatial learning and memory, anhedonic-like behaviors, and anxiety-like behaviors in male but not female rats, which raised the possibility that endogenous KL plays a key role in the sex differences in stress resilience. To address this question, endogenous KL expression in the rat hippocampus of both sexes was knocked down (KL-KD) by expressing the AAV vector encoding KL-shRNA for 3 weeks compared to the control rats of both sexes expressing scrambled shRNA (KL-CO; female: *t*_10_ = 29.9, *p* < 0.001; male: *t*_10_ = 6.46, *p* < 0.001; Figure 4A–D). 

Spatial learning and memory. The MWM test showed that KL-KD did not have a significant effect on the latency to the platform during training on days 1–5, the number of platform area crossings, and the time spent in the target quadrant on day 6 during the probe trail (Figure 4E–G, Table S1). 

Anhedonic-like and anxiety-like behaviors. KL-KD did not have a significant effect on sucrose preference in the sucrose preference test (Figure 4H), the time spent in the center of the open field (Figure 4I), the number of rearing times (Figure 4J), the number of grooming times (Figure 4K), and the total distance traveled in the OFT (Figure 4L) in rats of both sexes (Table S1). These results showed that the reduction in hippocampal KL does not impair spatial learning and memory and does not affect anhedonic-like and anxiety-like behaviors in rats of both sexes. The next experiment was to determine whether a reduction in endogenous KL levels alters the sex differences in stress resilience.

### 2.5. Endogenous KL Plays an Essential Role in Sex Differences in CUMS-Induced Deficit in Spatial Learning and Memory, Anhedonic-like Behaviors, and Anxiety-like Behaviors

**Figure 5:**
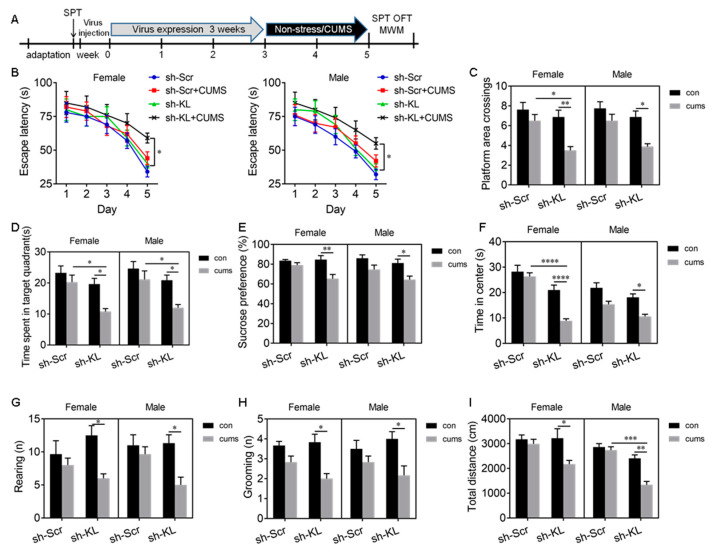
AAV-mediated decrease in KL protein levels increased stress susceptibility and diminished sex differences in spatial learning and memory deficit, anhedonic-like behaviors, and anxiety-like behaviors induced by 2-week CUMS. Experimental design. Sucrose preference test (SPT), open-field test (OFT), and Morris water maze (MWM) test (**A**). In the MWM test, CUMS did not alter the latency to the platform in KL normal rats of both sexes during 5-day training but CUMS caused a decrease in the latency to the platform in KL-KD (knockdown) rats of both sexes on day 5 during training (**B**). CUMS did not alter the number of platform area crossings (**C**) and the time spent in the target quadrant (**D**) in KL normal rats of both sexes. In contrast, in KL-KD rats of both sexes, CUMS decreased the number of platform area crossings (**C**) and the time spent in the target quadrant (**D**) in the MWM test. CUMS caused a decrease in sucrose consumption in the SPT (**E**), a decrease in the time spent in the center of the open field (**F**), a decrease in rearing times (**G**), a decrease in grooming times (**H**), and the total distance traveled (**I**) in the OFT in KL-KD rats of both sexes. Data are shown as the mean ± SEM. (**B**) Three-way repeated-measures ANOVA; (**C**–**I**) three-way ANOVA followed by Tukey’s test. * *p* < 0.05, ** *p* < 0.01, *** *p* < 0.001, **** *p* < 0.0001, n = 6–8. sh-KL, KL-shRNA; sh-Scr, control scrambled shRNA.

To determine whether KL-KD alters the stress resilience in female rats, rats of both sexes that had KL-KD or KL-CO were subjected to CUMS for 2 weeks (Figure 5A). The rationale for using 2-week CUMS was based on our time course of the sucrose preference test in which 2-week but not 1-week CUMS induced a significant decrease in sucrose consumption in KL-shRNA-expressing rats but not in scrambled-shRNA-expressing rats of both sexes. Three-week CUMS induced behavioral phenotypes in normal male rats only (Figure 3). 

Spatial learning and memory. Three-way ANOVA revealed a significant main effect of shRNA (*F*_1, 56_ = 10.99, *p* =0.002) and stress (*F*_1, 56_ = 25.61, *p* < 0.0001) on the escape latency on day 5 during training (Table S2). Tukey’s test showed that 2-week CUMS caused a significant decrease in the escape latency in KL-KD female (*p* < 0.05) and male (*p* < 0.05) rats compared to unstressed KL-CO controls (Figure 5B). However, CUMS did not alter the escape latency in normal rats of both sexes. These results showed that 2-week CUMS induces a deficit in spatial learning in male and female rats when endogenous KL expression reduces. Consistent with these results, there was a significant main effect of shRNA (*F*_1, 56_ = 18.17, *p* < 0.0001), a main effect of stress (*F*_1, 56_ = 26.47, *p* < 0.0001), and a significant effect of shRNA × stress interaction (*F*_1, 56_ = 5.53, *p* = 0.02) on platform area crossings (Figure 5C, Table S2). Tukey’s test showed that 2-week CUMS caused a significant decrease in platform area crossings in KL-KD females (*p* < 0.01) and males (*p* < 0.05) compared to unstressed KL-KD controls (Figure 5C). Similarly, there was a significant effect of shRNA (*F*_1, 56_ = 21.56, *p* < 0.0001), stress (*F*_1, 56_ = 18.75, *p* < 0.0001), and shRNA × stress interaction (*F*_1, 56_ = 4.04, *p* = 0.049) on the time spent in the target quadrant (Figure 5D, Table S2). CUMS caused a significant decrease in the time spent in the target quadrant in KL-KD females (*p* < 0.05) and KL-KD males (*p* < 0.05) compared to unstressed KL-KD controls (Figure 5D). However, CUMS did not alter these behaviors in normal KL-CO rats of both sexes. Two-week CUMS induced a similar deficit in spatial memory in KL-KD rats of both sexes, and reduction in endogenous KL expression diminished the sex differences in stress resilience. 

Anhedonic-like behavior. Three-way ANOVA showed that there was a significant main effect of shRNA (*F*_1, 56_ = 7.68, *p* = 0.008), stress (*F*_1, 56_ = 27.09, *p* < 0.0001), and shRNA × stress interaction (*F*_1, 56_= 4.1, *p* = 0.049) on sucrose consumption (Figure 5E, Table S2). Tukey’s test showed that CUMS caused a significant decrease in sucrose consumption in KL-KD females (*p* < 0.01) and males (*p* < 0.05) compared to unstressed KL-KD controls (Figure 5E). Two-week CUMS induced a similar level of anhedonic-like behavior in KL-KD but not normal male and female rats.

Anxiety-like behavior was evaluated by the time spent in the center of the open field. There was a significant main effect of shRNA (*F*_1, 56_ = 53.66, *p* < 0.001) and stress (*F*_1, 56_ = 38.05, *p* < 0.0001) and a significant effect of shRNA × stress interaction (*F*_1, 56_ = 6.14, *p* = 0.02) on the time spent in the center (Figure 5F, Table S2). Tukey’s test showed that CUMS caused a significant decrease in the time spent in the center in KL-KD females (*p* < 0.0001) and males (*p* < 0.05) compared to unstressed female and male KL-KD controls, respectively (Figure 5F). These results showed that 2-week CUMS induces anxiety-like behavior in KL-KD female and male rats, with a more severe phenotype observed in females. In addition, there was a significant effect of stress on rearing (*F*_1, 56_ = 17.11, *p* = 0.0002; Figure 5G) and grooming (*F*_1, 56_ = 26.55, *p* < 0.0001; Figure 5H) and a also significant effect of shRNA × stress interaction on rearing (*F*_1, 56_= 6.6, *p* = 0.01; Figure 5G) and grooming (*F*_1, 56_= 4.67, *p* = 0.04; Figure 5H, Table S2). Post hoc multiple-comparison tests revealed that CUMS caused a significant decrease in rearing (*p* < 0.05) and grooming (*p* < 0.05) times in KL-KD but not normal KL-CO rats of both sexes compared to unstressed controls (Figure 5G,H). CUMS induced a similar decrease in rearing and grooming times in male and female rats when endogenous KL expression reduced. Similarly, there was a significant main effect of shRNA (*F*_1,56_ = 22.3, *p* < 0.0001), stress (*F*_1, 56_ = 18.96, *p* < 0.0001), and shRNA × stress interaction (*F*_1, 56_ = 10.57, *p* = 0.002) on the total distance traveled (Figure 5I, Table S2). CUMS caused a significant decrease in the total distance traveled by KL-KD males (*p* < 0.05) and females (*p* < 0.01) but not by normal KL-CO males and females compared to unstressed controls (Figure 5I). These results showed that 2-week CUMS induces a similar decrease in the total distance traveled by KL-KD male and KL-KD female rats.

Overall, in KL-intact control rats of both sexes, 2-week CUMS did not alter cognitive function and did not induce anhedonic-like and anxiety-like behaviors. A reduction in KL expression in the hippocampus did not alter these behaviors but decreased the resilience, especially in female rats, to stress and diminished the sex differences in CUMS-induced deficits in spatial learning and memory, anhedoic-like behaviors, and anxiety-like behaviors, highlighting an important role of KL in the sex differences in stress resilience.

## 3. Discussion

### 3.1. E2 Regulated KL Expression in Hippocampal Neurons

Immunostaining showed that KL is localized in the soma and dendrites of hippocampal CA1 pyramidal neurons and MAP2-positive dendrites of cultured hippocampal neurons, in agreement with previous studies [5,31]. E2 treatments for 48 h and 7 days significantly reversed the OVX-mediated decrease in KL protein levels in the hippocampus. There are three KL forms with different functions in humans and mice: a full length, transmembrane KL (130 kDa), a shed KL, and a secreted KL (70 kDa) [6,7,41]. Our study focused on full-length KL. The secreted KL, which is expressed in mice and humans but not expressed in rats [10,11], plays an important role in spatial learning and memory in mice [6,42]. Our Western blot results confirmed that E2 replacement reverses the OVX-mediated decrease in KL protein (130 kDa) levels in the OVX rat hippocampus. This is in agreement with our previous study [4]. To the best of our knowledge, this is the first report that showed the regulation of KL protein by E2 in the rat hippocampus. E2 increases the excitatory synapse number in hippocampal neurons [33]. KL-deficient mice show a decrease in the synapse number in the hippocampus [13]. These studies suggest an important role of KL in E2-mediated synapse formation.

Our results indicated localization of KL-positive clusters on the dendrites of dissociated hippocampal neurons, and E2 treatment caused an increase in the number of both KL-positive and Vglut1-positive clusters along MAP2-positive dendrites. Vglut1 is localized to the presynaptic side of excitatory synapses, and the number of Vglut1-positive clusters on the dendrites reflects the number of excitatory synapses [32]. The E2-mediated increase in the number of Vglut1-postitive clusters is in agreement with our previous study [32] and reports by others [33,43,44]. Western blot analysis confirmed the upregulation of KL protein by E2 in dissociated hippocampal neurons. A reversal of the E2-mediated increase in KL protein levels in hippocampal neurons by ICI182,780 [33] showed the regulation of KL expression by E2 via estrogen receptors, but further study is required to determine which estrogen receptors contribute to this effect. 

### 3.2. KL Plays an Essential Role in E2-Mediated Synapse Formation in Hippocampal Neurons

The letrozole-mediated decrease in the number of KL-positive clusters was accompanied by a decrease in the number of Vglut1-positive clusters in the dendrites of rat hippocampal neurons, in agreement with a previous report indicating that the inhibition of endogenous E2 synthesis by letrozole causes a decrease in the synapse number in hippocampal neurons [43,45], which raised the hypothesis that KL plays an important role in E2-mediated synapse formation. E2 treatment caused a significant increase in the number of Vglut1-positive clusters along the MAP2-positive dendrites of GFP-expressing hippocampal neurons expressing scrambled shRNA. As expected, the ability of E2 to increase the number of Vglut1-positive clusters was eliminated in cultured hippocampal neurons expressing KL-shRNA, which showed that E2 treatment no longer significantly increased the number of Vglut1-positive clusters when endogenous KL expression in these neurons reduced. These results demonstrated that endogenous KL plays an essential role in the E2-mediated formation of excitatory synapses in hippocampal neurons. Importantly, our results showed that decreased KL levels due to KL-shRNA led to a similar decrease in the number of Vglut1-positive clusters in hippocampal neurons compared to scrambled-shRNA-expressing control neurons in which KL levels were intact. These results showed an essential role of KL in the formation of excitatory synapses in hippocampal neurons. The mechanism through which endogenous KL regulates the number of Vglut1-positive clusters and affects the E2-mediated formation of Vglut1-positive clusters is one of our future research directions. Estrogen plays a key role in the sex differences in cognition, and there is a sex difference in both stress resilience and chronic-stress-mediated cognitive deficits in rodents [29]. KL may play a role in sex differences in stress resilience characterized by sex differences in cognitive deficits and anhedonic-like and anxiety-like behaviors induced by CUMS.

### 3.3. Sex Differences in CUMS-Induced KL Expression Is Associated with Sex Differences in Stress Resilience in Behaviors

Cognitive deficit, anhedonia, and anxiety are often comorbid in depression [46,47]. CUMS, an established animal model of depression, is widely used to induce cognitive deficits, anhedonic-like behaviors, and anxiety-like behaviors in rodents [35,48]. CUMS-induced deficits in spatial learning and memory in male but not female rats are in agreement with previous studies in which chronic stress generally induced a cognitive deficit in male rats but did not have an effect on cognition or even enhance cognitive function in female rats [27,29]. The CUMS-induced decrease in the time spent in the center of the open field in males showed increased anxiety-like behavior, consistent with previous reports [49,50]. There are conflicting reports about the sex differences in CUMS-induced anhedonic-like behaviors in rats [51,52,53,54]. Rearing and grooming behaviors can be used as indicators of anxiety and emotionality in rats [55,56]. The CUMS-induced decrease in the times of both grooming and rearing led to a high level of anxiety-like behavior [57], in agreement with previous reports [58,59]. The CUMS-induced anhedonic-like behavior and the decrease in grooming and rearing times are consistent with the previous findings in animal models of depression [55,60]. The estrous cycle in female rats was not considered in this study as CUMS disrupts the estrous cycle by prolonging it [61,62]. The CUMS-mediated decrease in the level of KL protein in the hippocampus is consistent with previous reports in which chronic stress caused a decrease in the KL mRNA levels in the hippocampus and choroid plexus of male rats [20,63], but female rats were not included in these studies. However, the mechanisms through which chronic stress induces sex differences in cognitive deficit are largely unknown. In this study, the CUMS-induced deficit in spatial learning and memory, and anhedonic-like and anxiety-like behaviors in male rats was accompanied by a decrease in the levels of KL protein in the hippocampus of male but not female rats. Previous reports show that KL in the brain is required for maintaining normal cognitive function in mice, since global knockdown of KL in the mouse brain causes cognitive deficit [14]. The global overexpression of KL in the mouse brain [64] and KL overexpression in the bilateral ventricles of the mouse brain enhance cognition [65]. The downregulation of hippocampal KL is correlated with impairment in hippocampal-dependent memory in mice [66]. These results generated our hypothesis that the inability to decrease KL expression in the female hippocampus in the presence of chronic stress is responsible for the sex differences in chronic-stress-induced deficit in spatial learning and memory, and anhedonic-like and anxiety-like behaviors. We tested our hypothesis and discuss the results next.

### 3.4. Endogenous KL in the Hippocampus Is Essential for Sex Differences in Stress Resilience

To test our hypothesis, rats of both sexes were exposed to CUMS after knocking down the expression of full-length KL in the bilateral hippocampus. The rationale for selecting the hippocampus is that the hippocampus is vulnerable to stress, associated with anhedonic-like and anxiety-like behaviors, and plays an important role in learning and memory. Furthermore, KL is highly expressed in the rat hippocampus. To the best of our knowledge, we showed for the first time that the specific knockdown of KL in the rat hippocampus does not induce a clear deficit in spatial learning and memory, anhedonic-like behaviors, and anxiety-like behaviors in males and females. KL-deficiency-mediated impairment of cognition in mice may contribute to secreted KL [67] because overexpression of the secreted form of KL enhances cognition, while a reduction in the secreted form of KL impairs cognition in mice [42]. The secreted form of KL is expressed in the mouse but not rat hippocampus [11]. There are two KL forms in the rat hippocampus, a full-length protein (130 kDa) and a shed form [11]. Our KL-shRNA focuses on targeting the full-length form. Almost all of our knowledge about the function of KL in cognition was generated from mice. We did not see behavioral phenotypes after reducing the levels of full-length KL protein in the rat hippocampus, which may be the result of several reasons: (1) Rats do not express the secreted KL, (2) the amplitude of shRNA-mediated KL reduction in the hippocampus is not big enough to generate behavioral phenotypes, and (3) the differences in the genetic back between mice and rats may make a partial contribution. Our results suggest that full-length KL in the hippocampus does not play an essential role in maintaining cognitive function in rats, which requires further study. Interestingly, 3-week CUMS impaired spatial learning and memory in male but not female rats, as expected, when endogenous KL expression was not disrupted. However, 2-week CUMS induced a deficit in spatial learning memory, anhedonic-like behaviors, and anxiety-like behaviors in both female and male rats when endogenous KL levels reduced using KL-shRNA. Two-week CUMS did not alter cognitive function, anhedonic-like behaviors, and anxiety behaviors in the rats of both sexes when endogenous KL levels were intact. Furthermore, the sex differences in the CUMS-induced deficit in spatial learning and memory, anhedonic-like behaviors, and anxiety-like behaviors decreased when the levels of endogenous KL protein reduced in the rat hippocampus of both sexes. These results showed a key role of KL in sex differences in stress resilience. The question is how KL plays a key role in sex differences in stress resilience. KL regulates the formation of excitatory synapses. The CUMS-mediated decrease in the levels of KL in males may contribute to the CUMS-mediated decrease in spine density and synapse number [68]. Dendritic spines and synapses are both a cause and a consequence of behaviors, and spine density is correlated with behaviors and cognitive function [69,70] and stress resilience [71,72]. For example, the severity of depressive symptoms is correlated with low synapse density in the PFC of patients with MDD [73]. Therefore, the CUMS-mediated decrease in KL expression in males but not females contributes to sex differences in behavioral phenotypes induced by CUMS, which is a direction of our future study. Estrogen plays a key role in the sex differences in chronic-stress-mediated cognitive deficit, because inhibition of estrogen receptors or blocking of endogenous estrogen synthesis impairs cognition in female rats and diminishes the sex differences [27]. The underlying mechanisms are not clear. The regulation of KL by estrogen may contribute to the underlying mechanism. Our results indicated that E2 upregulates KL expression in the hippocampus of female rats that are resilient to CUMS-induced cognitive deficit, while a reduction in KL expression in the hippocampus decreases this resistance to stress in female rats and diminishes the sex differences in CUMS-induced deficit in spatial learning and memory, anhedonic-like behaviors, and anxiety-like behaviors by increasing the susceptibility of female rats to chronic stress. These studies suggest an important role of KL in sex differences in resilience to stress. Estrogen-mediated KL expression plays a crucial role in counteracting the detrimental effects of chronic stress on cognition in female rats. The underlying mechanism of this effect should be addressed in future studies. Full-length KL is expressed in rats and humans [7,11] and is regulated by E2 in the rat hippocampus. An enhanced understanding of the role of KL in stress responses, anxiety, anhedonia, and learning and memory in the rat model may enhance our understanding of the mechanisms underlying stress resilience, stress-related disorders, and learning and memory. It also has potential use in enhancing stress resilience and preventing stress-related disorders.

In conclusion, E2 upregulates KL protein levels in rat hippocampal neurons. E2 cannot increase Vglut1 expression and the number of Vglut1-positive clusters along the dendrites of hippocampal neurons when endogenous KL levels decrease. KL plays an essential role in the E2-mediated formation of excitatory synapses. A CUMS-mediated decrease in the level of KL protein in the hippocampus of male but not female rats is accompanied by a deficit in spatial learning and memory, anhedonic-like behaviors, and anxiety-like behaviors in males only. The AAV-KL-shRNA-mediated decrease in KL levels in the hippocampus of female rats decreases the resilience of female rats to stress and diminishes sex differences in CUMS-induced cognitive deficit, anhedonic-like behaviors, and anxiety-like behaviors. These findings support our hypothesis that KL plays a key role in sex differences in stress resilience.

## 4. Materials and Methods

### 4.1. Animals and Reagents

Ten-week old male and female Sprague–Dawley rats were purchased from the laboratory animal center of Xi’an Jiaotong University (Xi’an, China). The animals were housed in a temperature- (21 ± 1 °C) and humidity-controlled (60–65%) animal care facility with a 12 h light/12 h dark cycle and unrestricted access to food and tap water. The animal protocol was approved by the Animal Care Committee of Shaanxi Normal University (Xi’an, China). All manipulations were performed in accordance with the ethical principles of animal use and care. All animals were adapted to the laboratory conditions for 7 days before starting the experiment.

The following reagents were used: β-estradiol 3-benzoate (E8515, Sigma-Aldrich, St. Louis, MO, USA), sesame oil (S3547, Sigma-Aldrich, St. Louis, MO, USA), β-estradiol suitable for cell culture (E2257, Sigma-Aldrich, St. Louis, MO, USA), ICI182,780 (estrogen receptor antagonist, 1047, TOCRIS, Bristol, UK), letrozole (aromatase inhibitor, 4382, TOCRIS, Bristol, UK), and primary antibodies (Klotho (AF1819, R&D Systems, Minneapolis, MN, USA, immunostaining and Western blot), Klotho (ab203576, Abcam, Cambridge, UK Western blot), vesicular glutamate transporter (Vglut1, AB5905, Millipore, Bredford, MA, USA), and MAP2 (sc-32791, Santa Cruz Biotechnology, Dallas, TX, USA)).

### 4.2. Ovariectomy (OVX) and Estradiol Replacement

Bilateral OVX or sham surgery was performed, as described in our previous studies [4,32]. The animals were randomly divided into 4 groups: sham, OVX + vehicle, OVX + E2 (7 days), and OVX + E2 (48 h; n = 12, 6 for Western blot and 6 for immunohistochemistry). Ten days after surgery, OVX rats received a subcutaneous injection of 10 µg of β-estradiol 3-benzoate dissolved in 100 µL of sesame oil or 100 μL of sesame oil only (vehicle) [4]. For the OVX + E2 (7 days) group, the rats received E2 for 7 consecutive days, and for the OVX + E2 (48 h) group, rats received one subcutaneous injection of E2 and were sacrificed 48 h after injection. Successful OVX was confirmed by vaginal cytology, uterine wet weight, and circulating E2 levels, as described in our previous study [4].

### 4.3. Primary Rat Hippocampal Neurons, Drug Treatments, and Transfection

Primary cultures of hippocampal neurons were prepared from embryonic day 18 Sprague–Dawley rats of mixed sexes, as described previously [32]. The cultures were treated with 10 nM E2, the vehicle, E2 + 1 μM ICI182,780, or E2 + 0.1 μM letrozole at Div13 for 48 h, and then, the cultures were fixed with 4% paraformaldehyde and stained at Div15 with antibodies specific to KL, Vglut1, and MAP2, as described previously [34]. To reduce KL expression, two rat KL-shRNAs were designed (BLOCK-iT™ RNAi Designer). The shRNAs were annealed and ligated into a pAAV-U6-IRES-hrGFP vector. The inhibition efficiency of KL-shRNAs was confirmed in rat hippocampal cultures. One of the rat KL-shRNAs (CCTTACTTCGAGAAATGCGGG, nt1772-1792, NM_031336.1) was used based on its higher knockout efficiency, and the specificity of this shRNA was previously confirmed [74]. In addition, a non-target shRNA (scrambled shRNA; GTCTGTCCTGTCGTCTCTTAA) was used as a control. For shRNA knockdown experiments, the pAAV-U6-IRES-hrGFP vector encoding KL-shRNA or scrambled shRNA (control) was introduced into hippocampal neurons via electroporation at the time of plating at day 0 or using Lipofectamine 2000 reagent (Invitrogen, Carlsbad, CA, USA) at Div10, as described previously [32].

### 4.4. Western Blot

Western blot was performed, as described by Ma [75]. Total protein was extracted from the whole hippocampus or primary cultures using RIPA buffer (Solorbio Life Sciences, Beijing, China, # R0010). Samples were homogenized using a Pro Homogenizer, and the protein concentration was determined using the bicinchoninic acid assay (BCA) with bovine serum albumin as a standard. Briefly, 30 μg of proteins was loaded per lane on an 8–10% gradient acrylamide gel, and the proteins were transferred to Immobilon-*p* transfer membranes (Millipore) and incubated with the following primary antibodies: KL (1:1000, ab203576, Abcam) and GAPDH (1:10000, ZSGB-BIO). After incubation with the corresponding secondary antibodies, membranes were visualized using an luminescent imaging system (Tanon, China). The signal for each target protein was normalized to the GAPDH signal before being analyzed.

### 4.5. Immunohistochemistry (Brain Sections)

Immunohistochemistry was performed, as described in our previous report [32]. Briefly, coronal sections (12 µm) were cut in a cryostat and mounted on gelatin-coated slides. The sections were blocked in 1% BSA, 5% normal donkey serum, and 0.20% Triton X-100 (pH 7.4) for 1 h at room temperature. Next, the sections were stained with antibodies specific to KL at 4 °C overnight. Primary antibodies were visualized with Cy3-labeled donkey anti-rabbit IgG (Jackson Lab). Images were captured with a Zeiss LSM800 confocal microscope.

### 4.6. Immunocytochemistry of Cultured Hippocampal Neurons

Immunostaining of dissociated neurons was performed, as described previously by Ma et al. [32]. Briefly, neurons were fixed with 4% paraformaldehyde at room temperature for 18 min. After 7 min in 1% BSA, 5% normal donkey serum, and 0.20% Triton X-100, followed by 53 min in the same buffer without Triton X-100 at room temperature, the neurons were doubly stained with antibodies specific to KL or Vglut1 + MAP2 overnight at 4 °C. Primary antibodies were visualized with appropriate secondary antibodies [32]. Images were taken with a Zeiss LSM800 confocal microscope.

### 4.7. Chronic Unpredictable Mild Stress (CUMS)

CUMS was performed, as described by Qiao et al. [76] with a slight modification. Each rat was kept in one cage and was subjected to 8 unpredictable stressors, including (1) inversion of the light/dark cycle for 12/12 h, (2) swimming for 5 min in 4 °C cold water or 45 °C hot water, (3) a cage with damp sawdust for 24 h, (4) a cage tilting for 24 h, (5) shaking for 30 min, (6) tail nipping for 1 min, (7) fasting and water deprivation (overnight), and (8) lights on overnight. Rats received one unpredictable stressor per day in the first week, two stressors every other day in the second week, and two random stressors per day in the third week. The same stressor was not used in 2 consecutive days. Unstressed controls were simply handled for 2–3 min daily.

### 4.8. Stereotaxic Surgery and Adeno-Associated Virus (AAV) Microinjection

Stereotaxic surgery was performed, as described previously [77], according to the following coordinates: 3.0 mm posterior to the bregma, 2.2 mm lateral from the midline, and 2.4 mm below the skull surface. Next, 2 µL of AAV9 vectors encoding KL-shRNA or scrambled shRNA (packaged and purified by Hanbio Biotechnology, Shanghai, China) were injected into the bilateral hippocampus at an infusion rate of 0.2 µL/min using a micro-infusion pump. To prevent back-flow, the needle was left in place for an additional 10 min before it was slowly removed. After behavioral tests, we verified the accuracy of the injections by examining brain sections; animals were excluded from analysis when the injection site was off target.

### 4.9. Behavioral Assessments

Behavioral tests were performed 24 h after the last stress (Figure 3 and Figure 5) or 3 weeks after virus expression (Figure 4), and the order of the tests was the SPT, the OPT, and the MWM test.

The sucrose preference test (SPT) [76] was used as a measure of anhedonic-like behavior, a key symptom of depression [78]. For the sucrose preference test, controls and CUMS-exposed rats were deprived of food and water for 8 h (9:00–17:00) and subsequently exposed to 1% sucrose solution and water for 4 h from 17:00 to 21:00. The positions of the bottles were switched midway to prevent bottle preference. Sucrose and water consumption was measured, and the sucrose preference was calculated, as described previously [78]: sucrose preference index (%) = (sucrose intake/total fluid intake) × 100.

The open-field test (OFT) was conducted, as described in our previous study [78]. The time spent in the center of the open field was used to evaluate anxiety-like behavior. All rats were placed in the testing room for an hour before performing the OFT. The test was conducted for 5 min in a dimly lit room. Briefly, rats were placed in the center of the open-field box (60 cm long × 60 cm wide × 40 cm deep-black wooden box) individually, and the rats were allowed to explore the area freely during a 5 min test session. An automated video-based tracking system was used to record the locomotive activities of each rat during the test (Smart v3.0, RWD, Shenzhen, China). The total distance traveled, grooming and rearing times, and the time spent in the center of the open field were recorded. 

The Morris water maze (MWM) test was performed, as described with a slight modification [77]. The MWM test was used to evaluate spatial learning and memory. In the acquisition trials, rats received 4 training trials (60 s per trial) per day for 5 consecutive days and were trained to find the platform in one of four quadrants. Each rat was placed in a random starting location in the pool and given 60 s to swim to the platform in each trial. The rat was allowed to stay on the platform for 15 s. If the rat failed to reach the platform within 60 s, it was guided to the platform by the experimenter and was allowed to stay on the platform for 15 s. A probe trial was conducted 24 h after the 5-day training on day 6, with the hidden platform being removed. Rats were placed in a random starting location and allowed to swim to the former location of the hidden platform in the maze for 60 s. The latency to the platform in the acquisition trials, the number of crossings over the platform location, and the time spent in the target quadrant were recorded using a computerized video-based tracking system.

The Barnes maze test (BMT) was performed in an apparatus for rats, as shown in our previous study [79,80] with a minor modification. The BMT was used to evaluate spatial learning and memory. This test is generally thought to be less stressful than the Morris water maze test according to plasma corticosterone levels and more sensitive to early cognitive deficits [39,40]. The BMT consisted of three phases: (1) habituation, (2) training, and (3) testing. The rats were habituated to this maze by allowing them to freely explore the apparatus for 4 min or until they found an escape hole. Once the exploration period was over, each rat was gently guided to the escape hole compartment, where it remained for 1 min. The first training session was performed immediately after the habituation. During the training phase, the rats were trained to find the escape hole (target) for another 3 days (4 min/trial, 2 trials/day, 15–20 min apart between 2 trails). When a rat failed to find the escape hole within 4 min, a gloved hand was used to guide the rat to it. Once in the escape hole compartment, the rat was left there for 1 min before returning it to its home cage. The probe test (with the escape box removed) was conducted on day 7 after a 72 h break and carried out the same as the training trials. The latency to find the target hole, the time spent in the target quadrant, and the number of errors to locate the target were used to assess learning (during training) and memory (test on day 7) capabilities. Between trials, the surface was cleaned with 10% alcohol and then wiped dry. All trials were recorded on a video camera.

### 4.10. Data Collection and Statistical Analysis

All images were taken with identical settings under the same conditions using a Zeiss LSM800 confocal microscope and analyzed, as described previously [81]. Images were calibrated, and thresholds were set to ensure that all structures of interest were included in the analysis before performing analysis and counting synaptic clusters. Quantifications were performed using the MetaMorph image analysis system (Molecular Devices, Downington, PA, USA). Data are shown as the mean ± SEM. Inspection of the data did not reveal any departure from normality. Statistical analyses were performed with the *t*-test, one-way ANOVA, two-way ANOVA, three-way ANOVA, and repeated-measures two-way or three-way ANOVA (Figure 3B, Figure 4E and Figure 5B) followed by Tukey’s test. All the variables were all treatments/conditions, and then they were all fixed. All the computed *F*- and *p*-values of two-way ANOVA and three-way ANOVA are reported in the Supplementary Material (Tables S1 and S2). GraphPad Prism 8.0.2 was used for all analyses and drawing the graphs. *p* < 0.05 was defined as a significant difference.

## Data Availability

The data presented in this study are available in the article and Supplementary Materials.

## Supplementary Materials

The following supporting information can be downloaded at: https://www.mdpi.com/article/10.3390/ijms24021206/s1.
