# Supplementary material for: Klotho Regulated by Estrogen Plays a Key Role in Sex Differences in Stress Resilience in Rats
Source: Int J Mol Sci. 2023 Jan 7;24(2):1206. doi: 10.3390/ijms24021206 (PMC9862442; doi:10.3390/ijms24021206)
Supplement: Supplementary file 1 [file ijms-24-01206-s001.zip › ijms-2083959-supplementary.pdf]

Supplementary Materials

Summary of Supplementary Results

1. The specificity of Klotho (KL) antibody was validated in KL knockout mice (**Figure S1**). This antibody was used for immunostaining in this study.
2. Expression of Klotho was regulated by endogenous estrogen (E2) in cultured hippocampal neurons. Inhibition of endogenous E2 synthesis with letrozole caused a decrease in KL protein levels, KL positive clusters and Vglut1 positive clusters along MAP2 positive dendrites in the cultured hippocampal neurons (**Figure S2**).
3. Exogenous Estrogen (E2) increased the levels of Klotho protein expression in the hippocampus of male rats (**Figure S3**).
4. CUMS induced a deficit in spatial learning and memory in the Barnes maze test in male but not female rats, showing females are resilient to stress (**Figure S4**).
5. Summary of two-way ANOVA, three-way ANOVA (**Table S1-S2)**

Supplementary methods

Wildtype and KL knockout mice were fixed with 4% paraformaldehyde by perfusion. Fixation detail, immunostaining and western blot analysis were performed as described in the method section of the main text.

Hippocampal cultures prepared from embryonic day 18 rats of both sexes and quantification of KL positive and Vglut1 positive clusters were performed as described in the main text.

Chronic unpredictable mild stress (CUMS) in male and female rats was performed as described in the main text.

The Barnes maze test was performed as described in the main text.

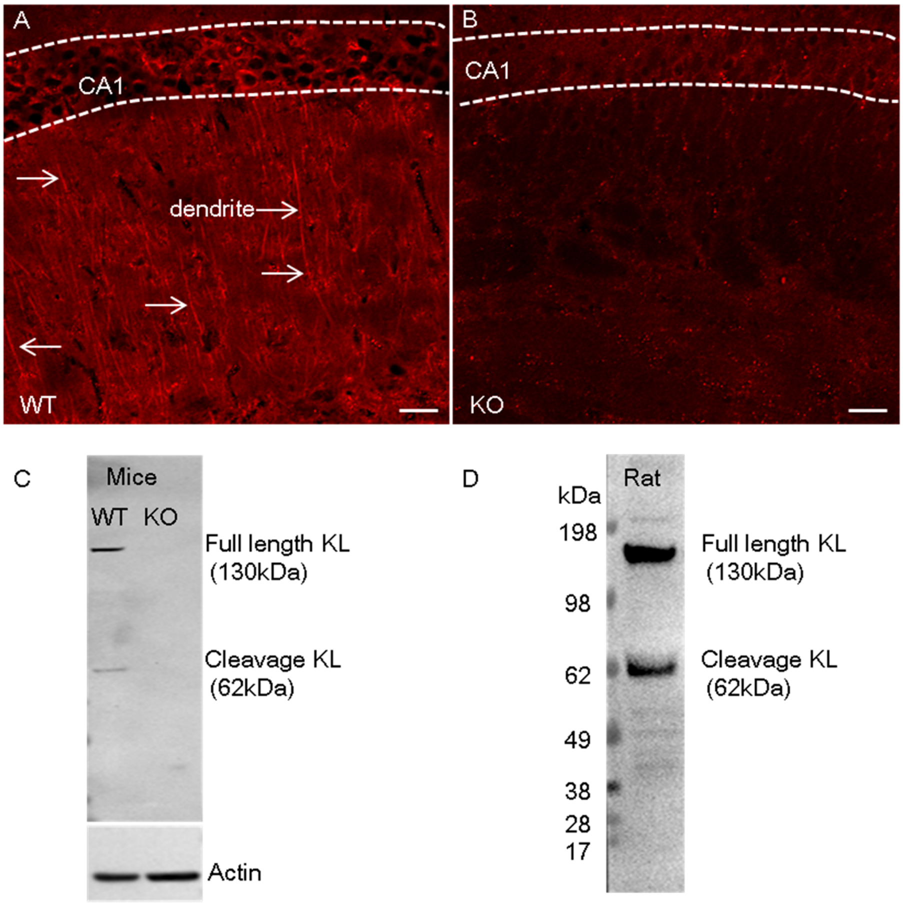

Figure S1. KL antibody that was used for immunostaining in this study is specific. Representative confocal images of a 2-month old wild-type (WT) mouse show that KL staining is localized (red) in the dendrites (white arrows) and soma of hippocampal CA1 pyramidal neurons (**A**), and KL staining in the same CA1 area of a KL knockout (KO) mouse is absent (**B**). Primary KL antibody was visualized with Cy3-labeled donkey anti-goat IgG (The Jackson Laboratory). Western blot shows that only specific KL protein bands are detected in the hippocampus of a 2-month old wild-type (WT) mouse, but not Klotho KO mouse (**C**). Western blot shows that only specific full length KL (130 kDa) band and cleavage KL band (62 kDa) are detected in the hippocampus of a 2-month old male rat (**D**). Scale bars = 20 μm.

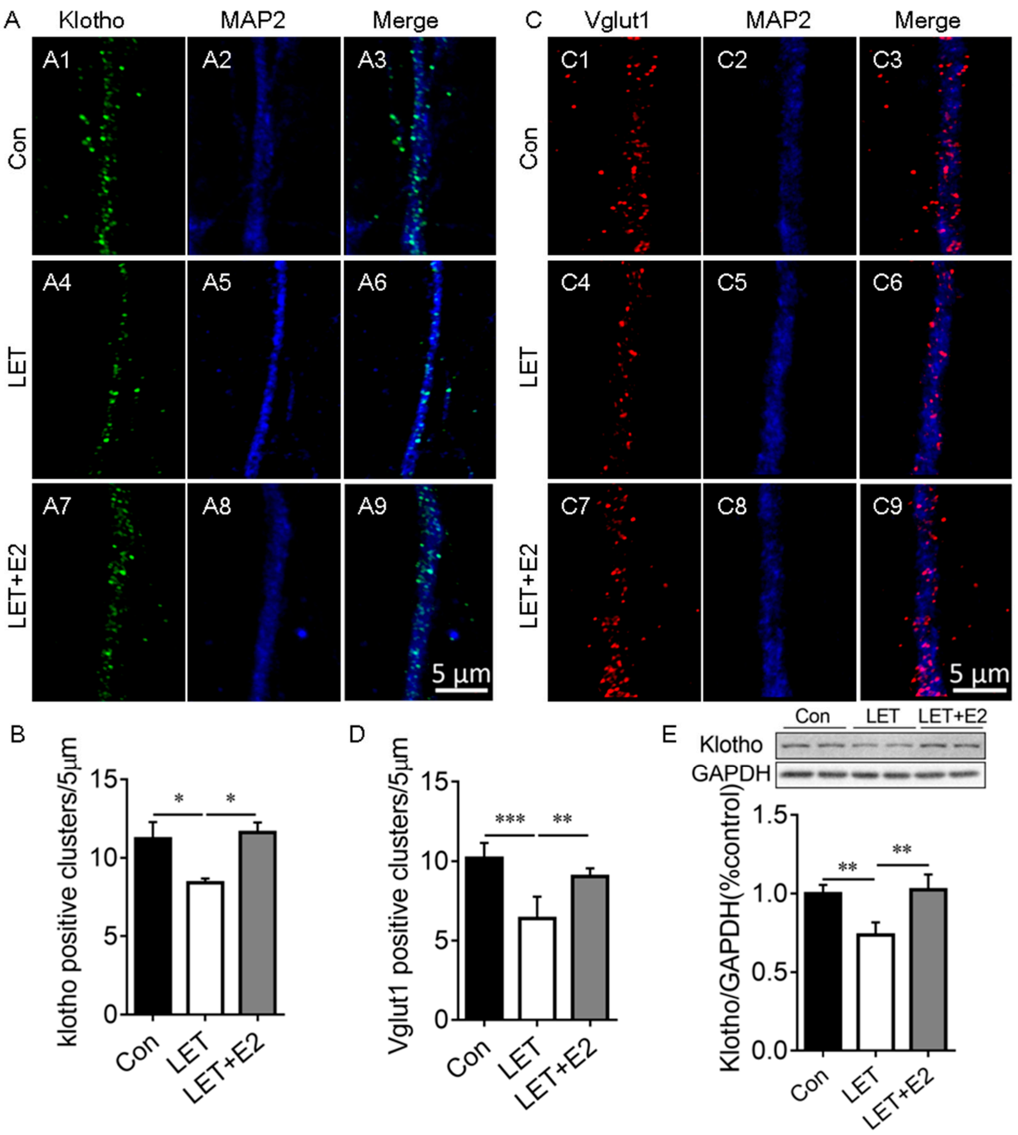

Figure S2. Expression of Klotho was regulated by endogenous estrogen (E2) in cultured hippocampal neurons. Hippocampal cultures prepared from embryonic *day* 18 rats of both sexes were treated with vehicle (control, Con), 0.1 μM letrozole (LET, aromatase inhibitor) and LET + E2 at Div 13 for 48 hours. Immunostaining of cultured neurons with antibodies specific to KL and MAP2 in Con, LET and LET+E2 treated neurons (**A**). Quantification of fluorescence intensity of KL staining in cultured hippocampal neurons (**B**). Immunostaining of cultured neurons with antibodies specific to Vglut1 and MAP2 in Con, LET and LET+E2 treated neurons (**C**). The number of Vglut1 positive clusters on MAP2 positive dendrites of Con, LET and LET+E2 treated neurons (**D**). Western blot analysis of KL protein in Con, LET and LET+E2 treated neurons (**E**). Data were shown as mean±SEM. One-way ANOVA followed by Tukey's test. $^*P < 0.05$, $^{**}P < 0.01$, $^{***}P < 0.001$. Scale bars = 5 μm.

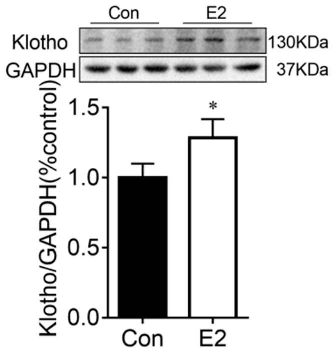

Figure S3. Estrogen (E2) increased the relative Klotho protein expression in hippocampus of male rats. After seven days of adaption to the laboratory conditions, male rats received subcutaneous injection of 10μg of β-Estradiol 3-benzoate dissolved in 100μl sesame oil (E2) or 100μl sesame oil only (Con) for seven consecutive days. Western blot result revealed that seven days of E2 treatment significantly increased the level of klotho protein in male hippocampus. Data were shown as mean±SEM. T-test, *$P < 0.05$, n= 6.

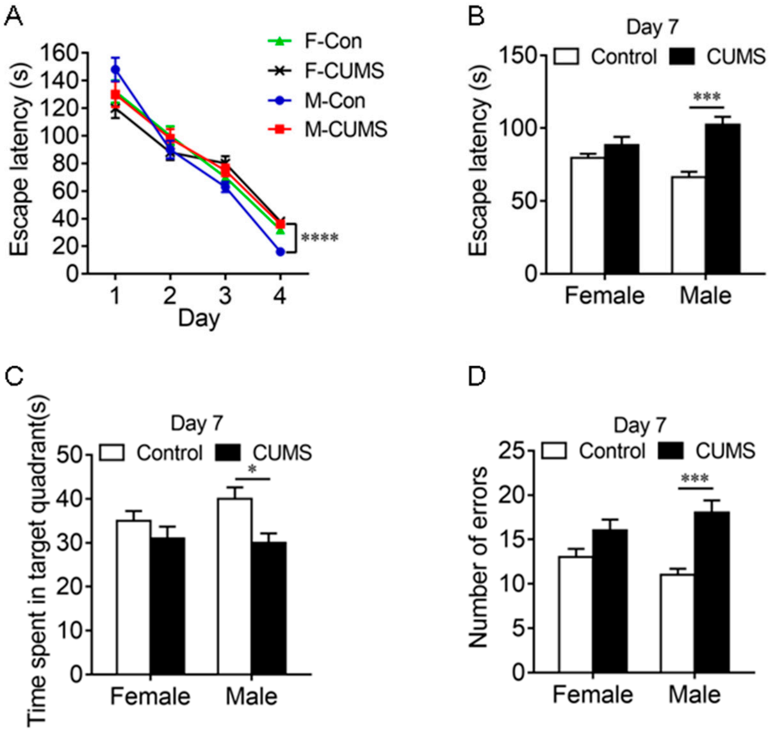

Figure S4. CUMS induced spatial learning and memory impairment in the Barnes maze test. The escape latency in rats of both sexes on day 1-4 during training phase in Barnes maze test (**A**) and the escape latency (**B**), the time spent in the target quadrant (**C**) and number of errors (**D**) to find target hole during probe trial on day 7. Data were shown as mean±SEM. Repeated measurement two-way ANOVA (A) or two-way ANOVA followed by Tukey's test. *$P < 0.05$, ***$P < 0.001$, ****$P < 0.0001$, n=7.

Table S1. Summary of Two-way ANOVA.

Fig 2	Parameter	shRNA x treatment		shRNA		treatment	
Fig 2E	Vglut1 positive clusters	$F_{1, 21} = 4.71$	$P = 0.04$	$F_{1, 21} = 324.8$	$P < 0.001$	$F_{1, 21} = 24.76$	$P < 0.001$
**Fig 3**	**Parameter**	**sex x stress**		**sex**		**stress**	
Fig 3B	Escape latency	$F_{1, 28} = 1.47$	$P = 0.2355$	$F_{1, 28} = 5.88$	$P = 0.022$	$F_{1, 28} = 23.51$	$P < 0.0001$
Fig 3C	Platform area crossings	$F_{1, 28} = 10.94$	$P = 0.003$	$F_{1, 28} = 3.29$	$P = 0.08$	$F_{1, 28} = 3.28$	$P = 0.08$
Fig 3D	Time spent in target quadrant	$F_{1, 28} = 9.29$	$P = 0.005$	$F_{1, 28} = 4.03$	$P = 0.05$	$F_{1, 28} = 12.97$	$P = 0.001$
Fig 3E	Sucrose preference	$F_{1, 36} = 1.76$	$P = 0.19$	$F_{1, 36} = 6.14$	$P = 0.02$	$F_{1, 36} = 9.15$	$P = 0.005$
Fig 3F	Time in center	$F_{1, 28} = 3.56$	$P = 0.069$	$F_{1, 28} = 32.05$	$P < 0.0001$	$F_{1, 28} = 8.89$	$P = 0.006$
Fig 3G	Rearing	$F_{1, 28} = 1.61$	$P = 0.21$	$F_{1, 28} = 6.31$	$P = 0.02$	$F_{1, 28} = 11.39$	$P = 0.002$
Fig 3H	Grooming	$F_{1, 28} = 0.04$	$P = 0.84$	$F_{1, 28} = 0.11$	$P = 0.74$	$F_{1, 28} = 4.25$	$P = 0.05$
Fig3I	Total distance	$F_{1, 28} = 1.72$	$P = 0.20$	$F_{1, 28} = 5.96$	$P = 0.02$	$F_{1, 28} = 9.98$	$P = 0.004$
Fig 3N	Integrated intensity of KL	$F_{1, 16} = 1.71$	$P = 0.21$	$F_{1, 16} = 0.34$	$P = 0.57$	$F_{1, 16} = 17.83$	$P = 0.006$
Fig 3P	KL protein expression	$F_{1, 24} = 7.13$	$P = 0.01$	$F_{1, 24} = 7.13$	$P = 0.01$	$F_{1, 24} = 18.16$	$P = 0.0003$
**Fig 4**	**Parameter**	**sex x shRNA**		**sex**		**shRNA**	
Fig 4E	Escape latency	$F_{1, 28} = 0.11$	$P = 0.74$	$F_{1, 28} = 5.60$	$P = 0.03$	$F_{1, 28} = 9.25$	$P = 0.005$
Fig 4F	Platform area crossings	$F_{1, 28} = 0.001$	$P = 0.97$	$F_{1, 28} = 1.28$	$P = 0.27$	$F_{1, 28} = 0.98$	$P = 0.33$
Fig 4G	Time spent in target quadrant	$F_{1, 28} = 0.16$	$P = 0.69$	$F_{1, 28} = 0.97$	$P = 0.33$	$F_{1, 28} = 7.11$	$P = 0.01$
Fig 4H	Sucrose preference	$F_{1, 45} = 0.04$	$P = 0.84$	$F_{1, 45} = 0.23$	$P = 0.64$	$F_{1, 45} = 1.67$	$P = 0.204$
Fig 4I	Time in center	$F_{1, 45} = 0.028$	$P = 0.87$	$F_{1, 45} = 9.38$	$P = 0.0048$	$F_{1, 45} = 3.18$	$P = 0.086$
Fig 4J	Rearing	$F_{1, 45} = 0.77$	$P = 0.38$	$F_{1, 45} = 6.03$	$P = 0.02$	$F_{1, 45} = 0.03$	$P = 0.86$

Fig4K	Grooming	$F_{1, 45} = 3.67$	$P = 0.06$	$F_{1, 45} = 3.18$	$P = 0.08$	$F_{1, 45} = 0.36$	$P = 0.55$
Fig4L	Total distance	$F_{1, 45} = 2.45$	$P = 0.12$	$F_{1, 45} = 3.14$	$P = 0.08$	$F_{1, 45} = 0.01$	$P = 0.92$
Fig S4	Parameter	sex x stress		sex		stress	
Fig S4A	Escape latency	$F_{1, 24} = 9.67$	$P = 0.0048$	$F_{1, 24} = 15.99$	$P = 0.0005$	$F_{1, 24} = 33.37$	$P < 0.0001$
Fig S4B	Escape latency (Day 7)	$F_{1, 24} = 8.71$	$P = 0.007$	$F_{1, 24} = 0.004$	$P = 0.9515$	$F_{1, 24} = 22.99$	$P < 0.0001$
Fig S4C	Time spent in target quadrant	$F_{1, 24} = 1.53$	$P = 0.23$	$F_{1, 24} = 0.68$	$P = 0.42$	$F_{1, 24} = 8.32$	$P = 0.0082$
Fig S4D	Number of errors	$F_{1, 24} = 3.294$	$P = 0.082$	$F_{1, 24} = 0$	$P > 0.99$	$F_{1, 24} = 20.59$	$P = 0.0001$

Table S2. Summary of Three-way ANOVA.

ANOVA table	F	P value
**Fig 5B Escape latency on day5**		
shRNA	$F_{1, 56} = 10.99$	P = 0.0016
sex	$F_{1, 56} = 1.096$	P = 0.2996
stress	$F_{1, 56} = 25.61$	P < 0.0001
shRNA x sex	$F_{1, 56} = 0.1218$	P = 0.7284
shRNA x stress	$F_{1, 56} = 2.467$	P = 0.1219
sex x stress	$F_{1, 56} = 0$	P > 0.9999
shRNA x sex x stress	$F_{1, 56} = 0$	P > 0.9999
**Fig 5C Platform area crossings**		
shRNA	$F_{1, 56} = 18.17$	P < 0.0001
sex	$F_{1, 56} = 0.08642$	P = 0.7699

stress	$F_{1, 56} = 26.47$	P < 0.0001
shRNA x sex	$F_{1, 56} = 0.0216$	P = 0.8837
shRNA x stress	$F_{1, 56} = 5.531$	P = 0.0222
sex x stress	$F_{1, 56} = 0.0216$	P = 0.8837
shRNA x sex x stress	$F_{1, 56} = 0.08642$	P = 0.7699
**Fig 5D Time spent in the target quadrant**		
shRNA	$F_{1, 56} = 21.56$	P < 0.0001
sex	$F_{1, 56} = 0.7195$	P = 0.3999
stress	$F_{1, 56} = 18.75$	P < 0.0001
shRNA x sex	$F_{1, 56} = 0.001993$	P = 0.9645
shRNA x stress	$F_{1, 56} = 4.036$	P = 0.0494
sex x stress	$F_{1, 56} = 0.007973$	P = 0.9292
shRNA x sex x stress	$F_{1, 56} = 0.007973$	P = 0.9292
**Fig 5E Sucrose consumption in the SPT**		
shRNA	$F_{1, 56} = 7.683$	P = 0.0084
sex	$F_{1, 56} = 0.4136$	P = 0.5238
stress	$F_{1, 56} = 27.09$	P < 0.0001
shRNA x sex	$F_{1, 56} = 0.06441$	P = 0.8010
shRNA x stress	$F_{1, 56} = 4.096$	P = 0.0497
sex x stress	$F_{1, 56} = 0.1828$	P = 0.6712
shRNA x sex x stress	$F_{1, 56} = 0.909$	P = 0.3461
**Fig 5F Time in center**		
shRNA	$F_{1, 56} = 53.66$	P < 0.0001
sex	$F_{1, 56} = 16.61$	P = 0.0001
stress	$F_{1, 56} = 38.05$	P < 0.0001
shRNA x sex	$F_{1, 56} = 12.82$	P = 0.0007
shRNA x stress	$F_{1, 56} = 6.143$	P = 0.0162
sex x stress	$F_{1, 56} = 0$	P > 0.9999

shRNA x sex x stress	$F_{1, 56} = 4.153$	P = 0.0463
**Fig 5G Rearing times**		
shRNA	$F_{1, 56} = 0.8362$	P = 0.3660
sex	$F_{1, 56} = 0.0474$	P = 0.8288
stress	$F_{1, 56} = 17.11$	P = 0.0002
shRNA x sex	$F_{1, 56} = 1.822$	P = 0.1846
shRNA x stress	$F_{1, 56} = 6.6$	P = 0.0140
sex x stress	$F_{1, 56} = 0.01706$	P = 0.8967
shRNA x sex x stress	$F_{1, 56} = 0.001896$	P = 0.9655
**Fig 5H Grooming**		
shRNA	$F_{1, 56} = 0.6906$	P = 0.4109
sex	$F_{1, 56} = 0.02762$	P = 0.8688
stress	$F_{1, 56} = 26.55$	P < 0.0001
shRNA x sex	$F_{1, 56} = 0.2486$	P = 0.6208
shRNA x stress	$F_{1, 56} = 4.669$	P = 0.0368
sex x stress	$F_{1, 56} = 0.02762$	P = 0.8688
shRNA x sex x stress	$F_{1, 56} = 0.02762$	P = 0.8688
**Fig 5I Total distance travelled**		
shRNA	$F_{1, 56} = 22.3$	P < 0.0001
sex	$F_{1, 56} = 15.8$	P = 0.0003
stress	$F_{1, 56} = 18.96$	P < 0.0001
shRNA x sex	$F_{1, 56} = 3.813$	P = 0.0579
shRNA x stress	$F_{1, 56} = 10.57$	P = 0.0023
sex x stress	$F_{1, 56} = 0.004789$	P = 0.9452
shRNA x sex x stress	$F_{1, 56} = 0.02266$	P = 0.8811
